# Ginsenoside Rg1 Inhibits Glucagon-Induced Hepatic Gluconeogenesis through Akt-FoxO1 Interaction: Erratum

**DOI:** 10.7150/thno.104739

**Published:** 2025-03-01

**Authors:** Qun Liu, Fei-Ge Zhang, Wen-Song Zhang, An Pan, Yi-Lin Yang, Jin-Feng Liu, Ping Li, Bao-Lin Liu, Lian-Wen Qi

**Affiliations:** 1 State Key Laboratory of Natural Medicines, China Pharmaceutical University, Nanjing 210009, China;; 2 Clinical Metabolomics Center, China Pharmaceutical University, Nanjing 211198, China.

The authors regret to find an error in the published version of Figure 4A, wherein the AKT band in HFD-fed mice was misused due to labeling errors during the figure compilation process. The authors had checked the original data and replaced the AKT band in the HFD-fed group in Fig. 4A with the correct one. The corrected version of Figure 4A is presented below. The corrections made in this erratum do not affect the original conclusions. The authors apologize for any inconvenience that the errors may have caused.

## Figures and Tables

**Figure 4:**
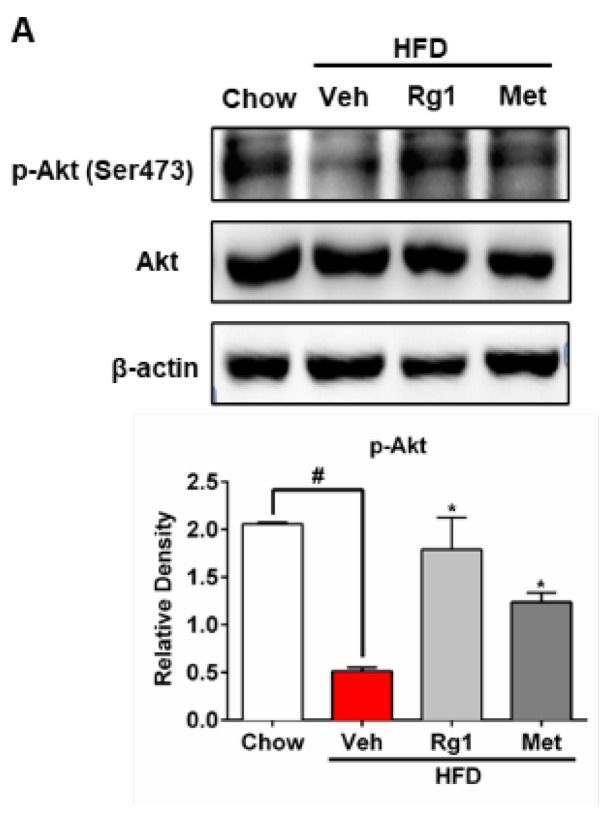
Correct image of Figure 4A.

